# *Legionella pneumophila* prevents proliferation of its natural host *Acanthamoeba castellanii*

**DOI:** 10.1038/srep36448

**Published:** 2016-11-02

**Authors:** Luce Mengue, Matthieu Régnacq, Willy Aucher, Emilie Portier, Yann Héchard, Ascel Samba-Louaka

**Affiliations:** 1 Laboratoire Ecologie et Biologie des Interactions, Microbiologie de l’Eau, Université de Poitiers, UMR CNRS 7267, Poitiers, France; 2 Signalisation et Transports Ioniques Membranaires, Université de Poitiers, CNRS ERL 7368, Poitiers, France

## Abstract

*Legionella pneumophila* is a ubiquitous, pathogenic, Gram-negative bacterium responsible for legionellosis. Like many other amoeba-resistant microorganisms, *L. pneumophila* resists host clearance and multiplies inside the cell. Through its Dot/Icm type IV secretion system, the bacterium injects more than three hundred effectors that modulate host cell physiology in order to promote its own intracellular replication. Here we report that *L. pneumophila* prevents proliferation of its natural host *Acanthamoeba castellanii*. Infected amoebae could not undergo DNA replication and no cell division was observed. The Dot/Icm secretion system was necessary for *L. pneumophila* to prevent the eukaryotic proliferation. The absence of proliferation was associated with altered amoebal morphology and with a decrease of mRNA transcript levels of CDC2b, a putative regulator of the *A. castellanii* cell cycle. Complementation of *CDC28*-deficient *Saccharomyces cerevisiae* by the *CDC2b* cDNA was sufficient to restore proliferation of *CDC28*-deficient *S. cerevisiae* and suggests for the first time that CDC2b from *A. castellanii* could be functional and a *bona fide* cyclin-dependent kinase. Hence, our results reveal that *L. pneumophila* impairs proliferation of *A. castellanii* and this effect could involve the cell cycle protein CDC2b.

*Legionella pneumophila* is a pathogenic Gram-negative bacterium found in natural and artificial aqueous environments^1^. It is responsible for legionellosis, a potentially lethal pneumonia that occurs after inhalation of aerosols containing the bacterium^2^. In water systems, *L. pneumophila* was found associated with free-living amoebae (FLA) which ensure its survival and replication^3^. The genus *Acanthamoeba*, among the most prevalent FLA found in the environment, has been characterized as a natural host of *L. pneumophila*^3^. The bacterium is phagocytized during *Acanthamoeba* grazing but it resists intracellular digestion^3^,^4^. *L. pneumophila* has evolved a number of mechanisms to modulate amoebal signal-transduction pathways to its advantage to support its replication. This successful strategy lies in the ability of *L. pneumophila* to perturb essential functions such as transcription, translation, cytoskeleton machineries, organelle function, vesicular trafficking, autophagy and host survival processes^5^. During infection of amoebae or alveolar macrophages, *L. pneumophila* injects more than three hundred effectors through a type IV secretion system (T4SS) called Dot/Icm (Defect in organelle trafficking; Intracellular multiplication) which is critical for resistance to host digestion, replication and exit of the bacterium from the cell^6^,^7^. The disturbance of eukaryotic functions by *L. pneumophila* is facilitated by bacterial proteins that contain a eukaryotic domain allowing the pathogen to mimic host cell functions^8^. To our knowledge, no study thus far has characterized the consequences of *L. pneumophila* infection on host-cell proliferation.

The cell cycle is a vital process that ensures growth and reproduction or proliferation of all living cells. It consists of duplication of the cell content (DNA and organelles) and repartition of the duplicated material into the daughter cells during mitosis and cytokinesis. The eukaryotic cell cycle is driven by a class of serine/threonine kinases named Cyclin-Dependent Kinases (CDKs) that act in concert with protein regulatory subunits called Cyclins^9^. Activities of some CDKs, such as the human protein CDK1 or the homologous protein in *Saccharomyces cerevisiae* CDC28, can be essential for cell proliferation^10^,^11^. Although CDK activity was detected in *Acanthamoeba*, the responsible protein had yet to be identified^12^. The sequencing of the genome of *A. castellanii* provided some clues as to the identity of the putative cell cycle regulator^13^.

Here, we show that *L. pneumophila* is able to prevent the proliferation of its natural host *Acanthamoeba*. This effect depends on the number of bacteria per amoebae and required the Dot/Icm secretion system. The arrest in *Acanthamoeba* multiplication provoked by *L. pneumophila* correlated with changes of the shape and motility of *Acanthamoeba*. Moreover, *L. pneumophila* induced a decrease in host mRNA levels of CDC2b, a putative CDK in *Acanthamoeba*. Furthermore, the genetic complementation of CDC2b cDNA from *Acanthamoeba* in *CDC28*-deficient *S. cerevisiae* demonstrates that CDC2b is a functional CDK and suggests that the cell cycle inhibition of *Acanthamoeba* upon infection could be related to down-regulation of CDC2b mRNA.

## Results

### *L. pneumophila* impairs proliferation of *A. castellanii*

**Figure 1:**
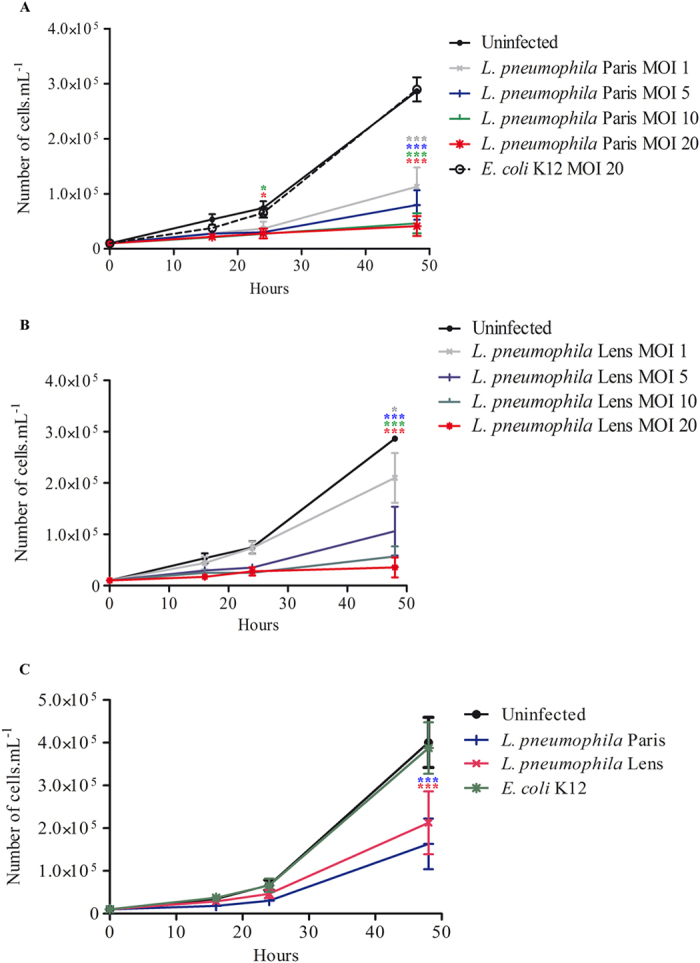
*L. pneumophila* prevents proliferation of *A. castellanii*. (**A**) *A. castellanii* ATCC 30010 were infected with *L. pneumophila* Paris and *E. coli* K12 or (**B**) with *L. pneumophila* Lens at a MOI of 1, 5, 10 and 20. Infections were carried out within the PAS solution for 2 h and cells were further incubated within the PYG medium containing gentamicin. Sixteen, twenty four and forty eight hours after infection, cells were harvested for counting. Results are average of three independent experiments and errors bars represent the standard error of the mean (±SEM). (**C**) *A. castellanii* ATCC 30234 were co-cultured with *L. pneumophila* Paris, *L. pneumophila* Lens and *E. coli* K12 at a MOI of 20. At different time points, cells were harvested for counting. Results are average of three independents experiments and errors bars represent the standard error of the mean (±SEM). The asterisks indicate conditions that are significantly different compared to uninfected cells (*p < 0.05; ***p < 0.001).

In order to assess the impact of *L. pneumophila* on amoebal proliferation, *A. castellanii* 30010 were infected with *L. pneumophila* Paris or with *Escherichia coli* K12, which does not resist host digestion, at multiplicities of infection (MOIs) of 1, 5, 10 and 20. Proliferation of *A. castellanii* was evaluated for 48 h. In contrast to uninfected cells or *A. castellanii* co-cultured with *E. coli*, we did not observe any increase of the cell population when *A. castellanii* was infected with *L. pneumophila* (Fig. 1A). The difference between *L. pneumophila* infected and uninfected cells was significant as soon as 24 h post-infection (Fig. 1A). The inhibition of cell proliferation upon infection with *L. pneumophila* seemed dependent on the MOI used as this effect was more pronounced with higher MOIs (Fig. 1A). To address the possibility that the absence of proliferation of *A. castellanii* induced by *L. pneumophila* was bacterial or amoebal strain specific, we infected *A. castellanii* 30010 with the *L. pneumophila* Lens strain. Although compared to uninfected cells the difference was less pronounced at a MOI of 1, *L. pneumophila* Lens also impaired proliferation of *A. castellanii* (Fig. 1B). Similarly, another strain of *A. castellanii* (ATCC 30234) was co-cultured with *L. pneumophila* (Lens and Paris strains) and with *E. coli* K12 at a MOI of 20. Less proliferation over time was observed when infection was performed with *L. pneumophila* compared to uninfected cells or those infected with *E. coli* K12 (Fig. 1C). According to these results, *L. pneumophila* prevents proliferation of *A. castellanii*.

### *L. pneumophila* inhibits proliferation of *A. castellanii* through the Dot/Icm secretion system

**Figure 2:**
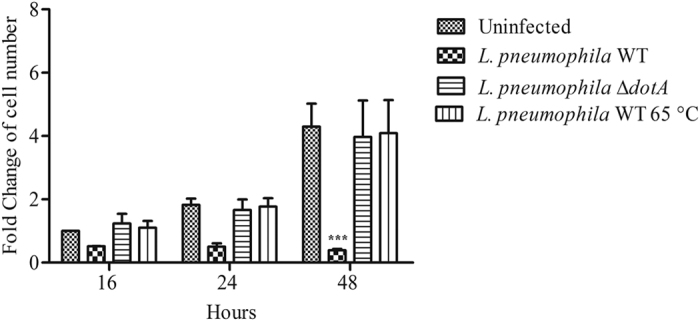
*L. pneumophila* prevents proliferation of *A. castellanii* through the Dot/Icm secretion system. *A. castellanii* ATCC 30010 were infected with wild type (WT) *L. pneumophila* Paris, heat-killed (65 °C) and ∆*dot*A *L. pneumophila* Paris at a MOI of 20. Sixteen, twenty-four and forty-eight hours after infection, cells were harvested for counting. Results are average of three independents experiments and errors bars represent the standard error of the mean. Cell numbers are relative to uninfected cells at 16 h. The asterisks indicate conditions that are significantly different compared to uninfected cells (***p < 0.001).

To address how *L. pneumophila* inhibited *A. castellanii* proliferation, amoebae were infected with live, heat-killed (65 °C) or ∆*dot*A mutant of *L. pneumophila* at a MOI of 20. The number of *A. castellanii* was assessed at different time-points to establish a kinetic of proliferation for each condition. In contrast to live *L. pneumophila*, infection with heat-killed or ∆*dot*A *L. pneumophila* did not impair proliferation of *A. castellanii* (Fig. 2). These results suggested that inhibition of *A. castellanii* proliferation requires live *L. pneumophila* with a functional Dot/Icm T4SS.

### *L. pneumophila* induces modifications in shape, motility and cell division of *A. castellanii*

**Figure 3:**
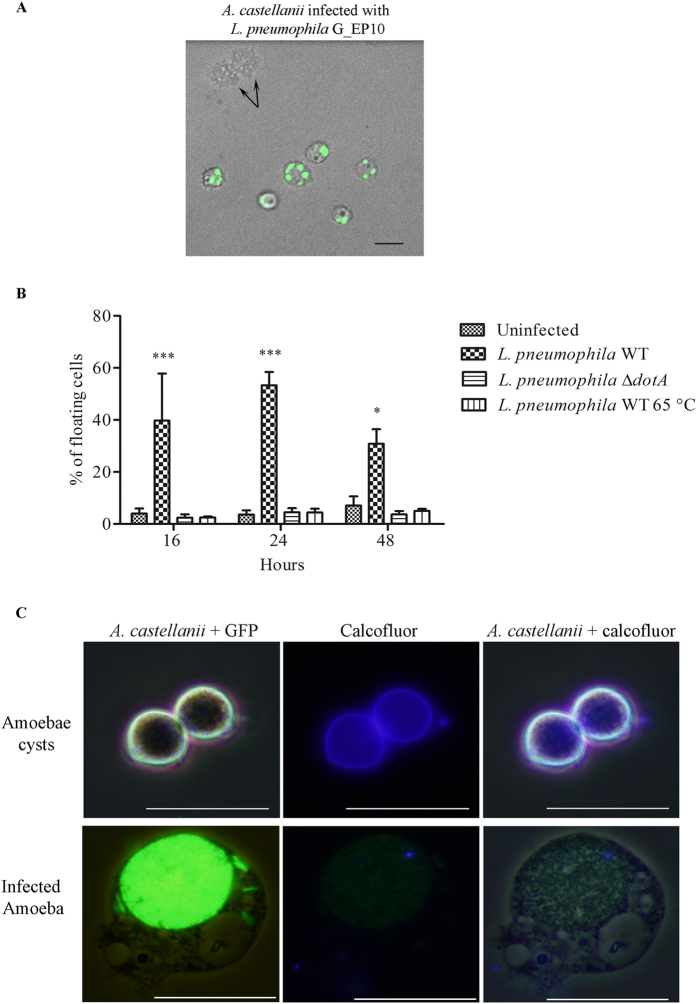
*L. pneumophila* modifies the shape of *A. castellanii.* (**A**) *A. castellanii* ATCC 30010 were infected with *L. pneumophila* Paris expressing GFP (G_EP10) at a MOI of 20. Green color represents the GFP signal. Images were captured 18 h post-infection. Black arrows show cell division of one mother cell into two daughter cells. The size bar represents 20 μm. (**B**) *A. castellanii* ATCC 30010 were infected with wild type (WT) *L. pneumophila* Paris, heat-killed and ∆*dot*A *L. pneumophila* Paris at a MOI of 20. At different time points, the percentage of floating cells was estimated. Results are average of three independents experiments and errors bars represent the standard error of the mean (±SEM). The asterisks indicate data that are significantly different compared to uninfected cells (*p < 0.05, ***p < 0.001). (**C**) *A. castellanii* ATCC 30010 were cultured in encystation medium or infected with *L. pneumophila* Paris G_EP10 at MOI of 20. Twenty-four hours after treatment or infection, cells were harvested and stained with calcofluor in order to reveal cyst forms. Transmitted light, green and blue channels represented respectively amoebae, GFP (*L. pneumophila*) and calcofluor (cellulose) signals. The size bar represents 20 μm.

Time lapse microscopy was performed to visualize cell division of *A. castellanii* after infection with *L. pneumophila* Paris expressing the GFP protein (G_EP10). Videos were performed 16 h after infection and taken for approximately 17 h. As shown in the supplementary video 1, uninfected *A. castellanii* were highly motile and cell division was observed. Whereas in infected cells, we did not observe any cell division in GFP positive cells (supplementary video 2 and Fig. 3A). Infected cells seemed less motile with modifications of their shape as they became rounded (supplementary video 2 and Fig. 3A). In addition, cells positive for GFP seemed less adherent than the GFP negative cells (supplementary video 2).

In order to test cell adherence of *A. castellanii* following infection with *L. pneumophila*, floating cells were harvested after infection with live, heat-killed or ∆*dot*A mutant of *L. pneumophila* at a MOI of 20. We observed that in contrast to heat-killed and ∆*dot*A *L. pneumophila*, live wild-type *L. pneumophila* seemed to induce cell detachment (Fig. 3B). These data indicate that inhibition of cell division induced by *L. pneumophila* was associated with modifications of the shape and of the motility of *A. castellanii*. Moreover, *L. pneumophila* required a functional Dot/Icm secretion system to decrease adherence of *A. castellanii* from the surface.

*A. castellanii* has a life cycle that oscillates between a dormant and a replicative forms named cyst and trophozoite, respectively. Under unfavorable conditions, amoebae form cysts, which are resistant to environmental stress and metabolically inactive^14^. During encystation, *A. castellanii* retracts its pseudopodia and becomes rounded^15^,^16^. In order to verify that rounded-infected cells were not encysted, *A. castellanii* were stained with Calcofluor White to reveal cellulose in the cell wall of mature cysts^17^. We found that cells that were highly infected with *L. pneumophila* G_EP10 did not share phenotypic characteristics with *A. castellanii* cysts. Indeed, there was a striking difference in cell size and, in contrast to cysts, no cell wall containing cellulose was observed in infected amoebae with *L. pneumophila* G_EP10 (Fig. 3C). In our experimental conditions, round cells that seemed barely adherent upon infection with *L. pneumophila* were not cysts.

### *L. pneumophila* impairs the ability of infected cells to replicate their DNA

**Figure 4:**
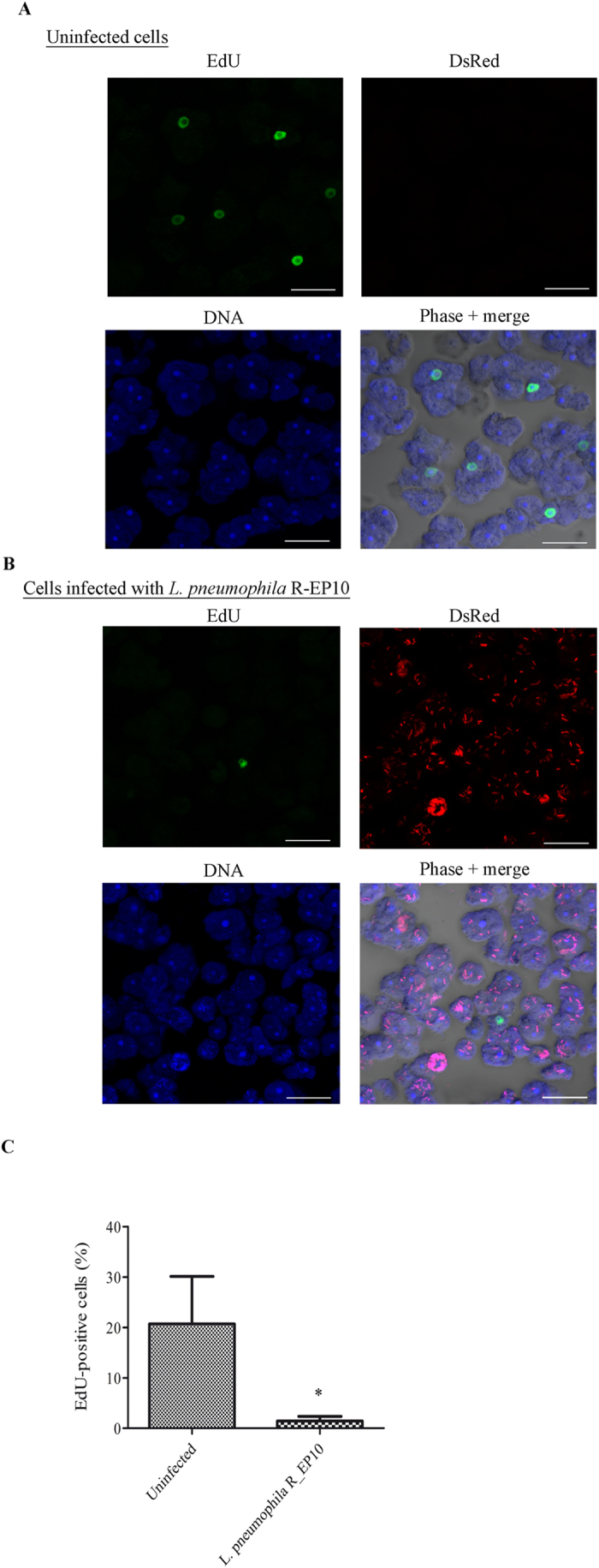
*L. pneumophila* prevents DNA synthesis in *A. castellanii*. (**A**) Uninfected *A. castellanii* 30010 and (**B**) cells infected at a MOI of 20 with *L. pneumophila* Paris expressing DsRed (R_EP10) were incubated in PYG medium supplemented with gentamicin (20 μg/ml) and EdU (40 μM) for 4 h. For infected cells, the EdU treatment started just after infection. Cells were harvested for microscopic observation. EdU appears in green, DsRed signal in red, nuclei in blue (TO-PRO^®^-3 Iodide). The size bar represents 20 μm. (**C**) Quantification of EdU positive cells from 3 independent experiments, for a total of more than 350 *A. castellanii* counted. The graph shows the mean +/− SEM (*p ≤ 0.05).

Cell division and DNA replication represent the two essential cell cycle phases of any eukaryotic cell. Thus we assessed whether the absence of cell division upon *L. pneumophila* infection was associated to an inhibition of DNA synthesis. *A. castellanii* infected or not with *L. pneumophila* Paris expressing the DsRed protein (R_EP10) were incubated in a growth medium containing 5-ethynyl-2′-deoxyuridine (EdU) for 4 h. Incorporation of EdU, an analogue of thymidine, indicates DNA synthesis. Thus, as expected, uninfected cells were able to duplicate their DNA since around 20% of cells were positive for the EdU signal (Fig. 4A,C). However, in *A. castellanii* that were co-cultured with *L. pneumophila* R_EP10, the number of cells positive for EdU dropped to around 1% (Fig. 4B,C). In addition, we never detected cells positive both for EdU and DsRed signals (Fig. 4B). This result suggests that *L. pneumophila* prevented DNA replication in *A. castellanii*.

### The protein CDC2b from *A. castellanii* is a putative CDK

**Figure 5:**
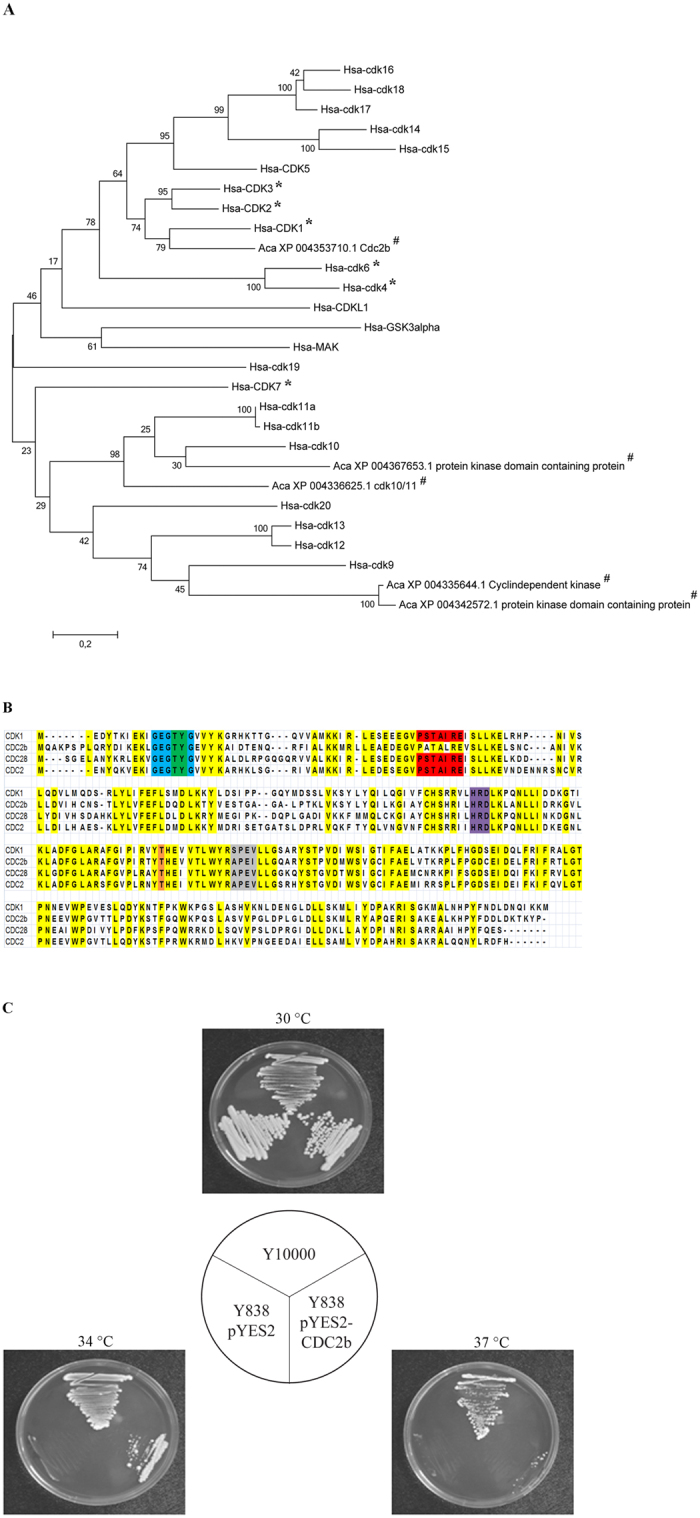
CDC2b from *A. castellanii* could be the regulator of the cell cycle. (**A**) CDK phylogeny inferred by Maximum Likelihood based on human (Hsa) CDKs^18^ and putative *A. castellanii* (Aca) CDKs. 1000 bootstrap replications were inferred and displayed at the nodes. The scale bar represents the number of amino acid substitution per site. All proteins were first labeled with their species name (Aca, *A. castellanii*; Hsa, *Homo sapiens*). *Indicates humans cell cycle-related proteins and # represents proteins from *A. castellanii*. (**B**) Sequence alignment between CDC2b (XP_004353710.1) from *A. castellanii,* CDK1 (NP_001777.1) from *Homo sapiens*, CDC28 (NP_009718.3) from *Saccharomyces cerevisiae* and CDC2 (CAC37513.1) from *Schizosaccharomyces pombe* using MUSCLE of the MEGA6 software. Yellow color highlight conserved residues. Motifs characteristics of CDK are displayed in blue (ATP-binding domain), green (inhibitory phosphorylation sites), red (cyclin-binding domain), purple (start of T-loop), orange (activating phosphorylation site) and grey (end of T-loop). (**C**) The *S. cerevisiae* strains Y10000 (wild type), Y838 (*cdc28-4*) transformed with the empty plasmid pYES2 or the recombinant plasmid pYES2-CDC2b were streaked on galactose-containing medium and cultivated at 30 °C, 34 °C or 37 °C for 4 days.

Eukaryotic cell cycle regulation is driven by cyclin dependent kinases (CDKs). Because *Homo sapiens* cells possess a considerable number of CDKs, we ran a blast search of protein sequences of human CDKs (supplementary Data 1)^18^ within the genome of *A. castellanii* (NCBI). Based on an E-value less than 1 and a percentage of identity of at least 35%, we obtained five candidates as putative CDKs of *A. castellanii* (Table 1). These five proteins were aligned with human CDKs in order to perform a phylogenetic analysis. Similarly to Cao *et al*.^18^, we used human (Hsa) cyclin-dependent kinase like-1 (CDKL1), glycogen synthase kinase-3 (GSK3) alpha and serine/threonine-protein kinase MAK isoform 1 as outgroups. Six of the human CDKs are directly related to the cell cycle (CDK1, CDK2, CDK3, CDK4, CDK6 and CDK7)^19^. As shown on the Fig. 5A, only the XP_004353710.1 CDC2b putative protein appeared phylogenetically similar to a cell cycle-related human CDK (CDK1).

In order to further assess the similarity between CDC2b from *A. castellanii* and well described eukaryotic CDKs, we aligned the protein sequences with those of human (CDK1), and of Baker’s and fission yeast, *Saccharomyces cerevisiae* (CDC28) and *Schizosaccharomyces pombe* (CDC2), respectively. We found that the CDC2b protein sequence was highly conserved compared to the CDKs from the other organisms (Fig. 5B). Aside from the PSTAIRE cyclin-binding domain, all the CDKs we aligned displayed many highly conserved residues and domains that characterize CDKs^19^ such as an ATP-binding domain, an inhibitory phosphorylation site, an activating phosphorylation site and the start and end of a T-loop (Fig. 5B). Thus, the putative protein CDC2b from *A. castellanii* shares strong homology with other CDKs that are essential for the progression of the cell cycle.

Since *S. cerevisiae* can be used to test the function of proteins from *A. castellanii*^20^, we sought to determine whether heterologous expression of CDC2b could complement the loss of function of the *CDC28* gene from yeast. To this end, the coding sequence of CDC2b was introduced into plasmid pYES2 under the control of an inducible *GAL1* promoter. The yeast strain Y838, which harbors a temperature sensitive mutation *cdc28-4*, was transformed with the recombinant plasmid pYES2-CDC2b or the empty vector as a negative control. After propagation of the transformants at the permissive temperature, they were challenged for growth on YPGal medium at various temperatures. As shown in Fig. 5C, Y838 transformed with pYES-CDC2b was capable of robust growth at 30 °C or 34 °C but could not grow at 37 °C, whereas the same strain transformed with the empty plasmid was unable to grow above 30 °C. In contrast, the wild type strain Y10000 was able to grow regardless of the temperature of incubation (Fig. 5C). We conclude from this experiment that *A. castellanii* CDC2b can restore growth of the *cdc28-4* mutant but not as well as a wild type yeast strain.

### *L. pneumophila* provokes a decrease of the *CDC2b* gene expression

**Figure 6:**
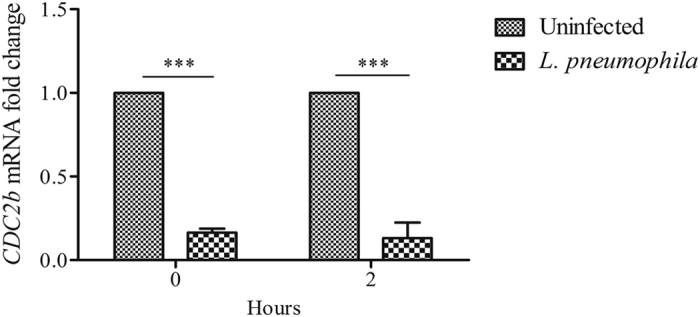
*L. pneumophila* induces a down-regulation of the *A. castellanii* cell cycle related protein CDC2b. *A. castellanii* ATCC 30010 were infected with *L. pneumophila* Paris at a MOI of 20. RT-qPCR for *CDC2b* mRNA extracted was performed just after the end of infection (0 h) and 2 h latter (2 h). Expression levels were normalized to the 18S mRNA. *CDC2b* levels are presented as levels relative to the uninfected condition. Results are average of three independent experiments and errors bars represent the standard error of the mean (±SEM). The asterisks indicate a significant difference between uninfected and infected conditions (***p < 0.001).

We next explored whether *L. pneumophila* modulates expression levels of CDC2b. We infected *A. castellanii* with *L. pneumophila* and monitored the level of CDC2b mRNA at different time-points. One found that *L. pneumophila* decreased CDC2b mRNA level as soon as 2 h after the beginning of the infection (0 h). The difference between uninfected and infected cells was significant at 0 and 2 h post-infection (Fig. 6). These results indicate that *L. pneumophila* infection leads to down-regulation of the mRNA levels of CDC2b, which correlates with a defect in amoebal proliferation during infection.

## Discussion

*L. pneumophila* is an intracellular bacterium that manipulates host functions to support its replication. However, the impact of *Legionella* infection on the host cell cycle has never been addressed. In this study, we demonstrate that *L. pneumophila* prevents multiplication of its natural host *A. castellanii* through the T4SS. Infection with *L. pneumophila* impairs cell division, DNA synthesis and motility of *A. castellanii.* The inability of infected cells to multiply correlates with a decrease of the mRNA levels of the putative amoebal cell cycle regulator CDC2b.

Some studies previously suggested a negative effect of *L. pneumophila* on the proliferation of *Acanthamoeba*^21^,^22^. In both studies, authors observed a decrease of the amoebal cell number upon infection, at least three days later, that was associated to an amoebal lysis^21^,^22^. Indeed, *L. pneumophila* provokes necrosis-mediated lysis of *Acanthamoeba* within 48 h after infection^23^. Here, we demonstrate that, even only several hours after infection with *L. pneumophila, A. castellanii* were unable to replicate their DNA, nor to undergo cytokinesis; two critical steps for cell proliferation. Thus, inhibition of proliferation, and not only *Legionella*-induced host lysis could account for this decrease in cell number. The type IV secretion system Dot/Icm was necessary for the inhibition of the host proliferation induced by *L. pneumophila*. Our ongoing work aims at identifying the *L. pneumophila* effectors involved in the regulation of proliferation of *A. castellanii*.

Our video microscopy experiments revealed that *L. pneumophila* induced a modification of the shape of amoebae which became rounded with some detachment from the substrate. The membrane of infected amoebae differed from mature *Acanthamoeba* cyst as no evidence for cellulose was observed by calcofluor stain. Modulation of encystment has been shown with other bacteria^24^,^25^. Regarding *Legionella*, controversially results could be found. Several papers have described cysts fully bound with *L. pneumophila*^26^,^27^ while some authors did not succeed to isolate infected cysts^28^. This suggests that some specific conditions might be required to obtain infected cysts with *L. pneumophila*. This question is of importance since cysts are thought to play a role in persistence and dissemination of *L. pneumophila*.

The alteration in amoebal shape and motility that we observed support previous results indicating that *L. pneumophila* modulates the cell host cytoskeletal molecules and migration^29^,^30^,^31^,^32^. Interestingly, treatment of the social amoeba *Dictyostelium discoideum* with the *Legionella* quorum sensing molecule LAI-1 induced down-regulation of genes involved in movement and in cell proliferation^31^. LAI-1 led to inactivation of the cytoskeletal-regulating protein CDC42 that was reported to induce cell cycle progression^33^,^34^. We aim to characterize the possible contribution of LAI-1 to the inhibition of *A. castellanii* proliferation in future studies.

*Legionella pneumophila* was shown to regulate transcription of genes implicated in proliferation of human monocyte-derived macrophages and of bone marrow-derived macrophages^35^,^36^. Furthermore, *L. pneumophila* modifies the host chromatin resulting in a repression of gene expression^37^,^38^. Since chromatin structure impinges on the cell cycle and vice-versa^39^, contribution of effectors implicated in chromatin-remodeling is a promising area to investigate.

To our knowledge, CDK proteins have never been characterized in *A. castellanii*. Thanks to genome sequencing of *A. castellanii*^13^, we found that the putative protein CDC2b shares a strong homology with other cell cycle-related CDKs. CDC2b could restore the ability of CDC28-deficient *S. cerevisiae* to grow although growth of transformants was limited at high temperature. Interestingly, the same conclusion was reported for the *CDC*2 gene of the distantly related amoeba *Dictyostelium discoideum*^40^. As CDK acts in concert with cyclin proteins, growth limitation of transformants carrying CDC2b could arise from the putative cyclin-binding domain of CDC2b that is different from the conserved PSTAIRE motif. This would be analogous to CDC2 from *D. discoideum* whose PSTAIRE is not completely conserved with an isoleucine substituted for leucine^40^.

From Amoebozoa to Vertebrata, several CDK proteins are present in one cell. The number of CDKs increases with eukaryotic evolution. While 20 CDKs are found in humans, only 6 and 8 CDKs are found in the yeast *S. cerevisiae* and in the amoeba *D. discoideum,* respectively^18^. As reported in yeast and mammalian systems, only one CDK is essential to drive the cell cycle from DNA replication to mitosis^10^,^11^. The ability of CDC2b to functionally complement CDC28-deficient *S. cerevisiae* suggests that CDC2b could represent the main cell cycle regulator in *A. castellanii*. Although as yet there is no method to generate mutants in *A. castellanii*, such experiments are needed to confirm our hypothesis.

Intracellular bacteria were reported to affect the growth rate of free-living amoebae^41^. Beyond, several bacteria are able to promote or to inhibit the eukaryotic cell cycle by using bacterial effectors called cyclomodulins^42^,^43^. Inhibitory cyclomodulins impair the function of cyclin/CDK complexes through activation of the DNA-damage response, tubulin protein sequestration, down-regulation of cyclins, stabilization of CDK inhibitors, interaction with proteins of the anaphase promoting complex or cleavage of the regulator of the endoplasmic reticulum (ER) function Bip^44^,^45^,^46^. Because cyclomodulins induce pleitropic effects on target cells, the next challenge will be to confirm that the decrease of CDC2b mRNA by *L. pneumophila* is responsible for the *A. castellanii* proliferation prevention and how this transcriptional/translational effect is important for *L. pneumophila*. Since down-regulation of CDK expression upon bacterial infection has never been reported, the down-regulation of the CDC2b mRNA level could be a novel strategy for a bacterium to regulate proliferation of its host.

In summary, this study shows for the first time that *L. pneumophila* prevents the proliferation of its host. Our work identifies a novel CDK of *A. castellanii* and shows that *Legionella* perturbs host proliferation, DNA replication and amoebal morphology during infection. Regulation of the host cell cycle contributes to the impressive list of the eukaryotic functions that are perturbed by *L. pneumophila*. We are currently attempting to decipher the signaling pathway responsible for *A. castellanii* cell cycle arrest and the bacterial effectors which mediate this effect during infection.

## Methods

### Strains, media and culture conditions

*A. castellanii* ATCC 30010 and ATCC 30234 were cultured in Peptone Yeast Glucose medium (2% proteose peptone, 0.1% yeast extract, 0.1 M glucose, 4 mM MgSO_4_, 0.4 mM CaCl_2_, 0.1% sodium citrate dehydrate, 0.05 mM Fe(NH_4_)_2_(SO_4_)_2_ 6H_2_O, 2.5 mM NaH_2_PO_3_, 2.5 mM K_2_HPO_3_, pH 6.5) at 30 °C.

To induce encystation, *A. castellanii* were incubated in encystation buffer (0.1 M KCl, 8 mM MgSO_4_, 0.4 mM CaCl_2_, 20 mM Tris (2-amino-2hydroxymethyl-1,3-propanediol), 1 mM NaHCO_3_, pH 8.8) at 30 °C for 24 h^47^.

*L. pneumophila* strains were cultured on buffered charcoal yeast extract (BCYE) (1% ACES, 1% yeast extract, 0.2% charcoal, 1.5% agar, 0.025% Iron (III) pyrophosphate, 0.04% L-cysteine, pH 6.9) agar plate. For *L. pneumophila* Paris strains, 0.1% alpha-ketoglutarate was added to medium. Bacteria were cultured on BCYE at 37 °C for 3 days. Then, bacteria were inoculated at the optical density at 600 nm (OD_600_) of 0.1 in Buffered Yeast Extract (BYE) at 37 °C under agitation 160 rpm to reach OD_600_ above 2.3.

Different strains of *L. pneumophila* were used as *L. pneumophila* Paris CIP 107629T^8^ and *L. pneumophila* Lens CIP108286^8^. The ∆*dot*A mutant of *L. pneumophila* Paris (resistant to the kanamycin (15 μg/mL)) was a gift of Carmen Buchrieser from Institut Pasteur (Paris, France)^48^. *E. coli* K12 (ATCC 10798) was cultured on Lysogeny Broth (LB) medium at 37 °C under agitation at 160 rpm.

*S. cerevisiae* strains were cultured in minimal YNB medium (yeast nitrogen base with ammonium sulfate and 2% glucose) supplemented with the appropriate amino acids and bases, or complete YPGal medium (1% yeast extract, 1% peptone, 2% galactose) at 28 °C unless otherwise indicated. Yeast strains used in this study are Y10000 (BY4742; *MATαura3Δ0leu2Δ0 his3Δ1 lys2Δ0*) and Y838 (*cdc28-4 ura3-52 lys2-801 leu2∆1 his3∆200*).

### Bacterial transformation

*L. pneumophila* Paris in exponential phase were washed with 10% cold glycerol solution and centrifuged (6000 g, 10 min at 4 °C). Pellets were washed in 20, 10 and 5 mL of 10% cold glycerol solution. Bacteria were re-suspended in 10% cold glycerol solution to reach an OD_600_ of 100. Electroporation was performed with the plasmid pSW001^49^ for bacteria producing the DsRed Fluorescent Protein and with pNT28 built from the plasmid pMMB207-Km14-GFPc^50^ for bacteria producing the Green Fluorescent Protein (GFP). One μg of plasmid and 100 μL of bacterial suspension were mixed. Electroporation was performed by using the EC2 programm (2.50 kV, 1 pulse) of the MicroPulser apparatus (Biorad). After the electroporation, 900 μL of liquid BYE were added to the bacteria followed by an incubation at 37 °C without agitation for two hours. Suspension was spread on BCYE supplemented with chloramphenicol (5 μg/mL) and the petri dishes were incubated at 37 °C during 3 or 4 days. The bacteria *L. pneumophila* Paris producing the DsRed and the GFP proteins were named *L. pneumophila* R_EP10 and *L. pneumophila* G_EP10 respectively.

### Infection of *A. castellanii*

Three days old *A. castellanii* were seeded in 24-well plates at 1 × 10^4^ cells per well in Page’s Amoeba Saline solution (PAS) (4 mM MgSO_4_, 0.4 M CaCl_2_, 0.1% sodium citrate dehydrate, 2.5 mM NaH_2_PO_3_, 2.5 mM K_2_HPO_3_, pH 6.5) for about 2 h at 30 °C. Once cells adhered on the plate, bacteria that reached an OD_600_ above 2.3 were added to the amoebae at different multiplicity of infection. The infection was synchronized by centrifugation (500 g, 10 min at room temperature). Co-cultures were incubated 2 h at 30 °C, rinsed with PAS and cultured within the PYG medium containing gentamicin (20 μg/mL). Plates were incubated at 30 °C.

To estimate the proliferation of *A. castellanii*, cells were harvested at different time-points and counted in triplicate for each condition using plastic counting slides FastRead 102 (Biosigma).

To obtain heat-killed *L. pneumophila*, bacteria were heated at 65 °C for 15 min.

Estimation of the cell detachment upon infection was performed by counting floating and adherent cells. The percentage of floating cells represents the number of floating cells under total cells, multiplied per 100.

Percentage of infected cells was assessed on epifluorescence microscope (Olympus BX41) using *L. pneumophila* Paris G_EP10. *A. castellanii* were seeded in 6-well plates at 1 × 10^6^ cells/well and infection was performed with *L. pneumophila* Paris G_EP10 at MOI 20. After the infection, cells were incubated 30 min in PYG medium, and amoebae were harvested, centrifuged (500 g, 5 min at room temperature) and washed with Phosphate-Buffered Saline (PBS). Cells were fixed for 15 min with 4% paraformaldehyde at room temperature, washed twice with PBS and centrifuged. The pellet was suspended in 40 μL of CitiFluor^®^ AF1 (Citifluor) and slide were analyzed under the microscope.

Cysts were assessed by a fluorescent microscopic detection using Calcofluor White Reagent Droppers (Becton Dickinson) according manufacturer’s instructions. 4.5 μL of *A. castellanii* and 0.5 μL of calcofluor were loaded on a glass slide. The mix was incubated 2 min at room temperature for staining followed by microscopic observations.

### Video microscopy

*A. castellanii* 30010 (1.35 × 10^4^ cells) were infected with *L. pneumophila* Paris G_EP10 at a MOI of 20 for 2 h in a μ-Slide 8 well IbiTreat microscopy chamber (Ibidi). Transmission images were acquired with confocal spinning disk from Andor technology mounted on an Olympus inverted IX81 microscope using Andor Ixon + 897 back illuminated EMCCD camera. Live imaging started 16 h after the end of the infection and run for around 17 h using the spinning disk confocal laser microscopy. Time lapse imaging was realized in 30 °C incubation chamber with x40 objective (512 × 512 pixels, 0.33 μm/pixel) and 1 image every 30 seconds.

### DNA synthesis

The DNA synthesis was determined using the Click-iT^®^ EdU Imaging Kits (Invitrogen^™^) following manufacturer’s recommendations with some modifications. Briefly, 1 × 10^6^ *A. castellanii* cells were infected with *L. pneumophila* Paris R_EP10 at MOI 20 in 6-well plates. After infection period, EdU (40 μM) was added to the PYG medium supplemented with gentamicin (20 μg/mL) for 4 h. Amoebae were harvested and centrifuged (1000 g, 10 min at room temperature) before fixation for 15 min at room temperature with 3.7% paraformaldehyde. Amoebae were washed twice with 1 mL of washing solution (3% bovine serum albumin (BSA) (Sigma)) in PBS and lysed for 20 min at room temperature with 1% Triton^®^ X-100 (Sigma). After two additional washes, 0.5 mL of Click-iT^®^ reaction cocktail (according to the manufacturer’s instructions) was added to the pellet and incubated 1 h protected from the light. For DNA staining, amoebae were washed with 1 mL of PBS, treated with TO-PRO^®^-3 Iodide (dilution of 1:1000) and incubated for 1 h at room temperature in the dark. Amoebae were washed for the last time with PBS and suspended in washing buffer for analysis.

*A. castellanii* were examined with a laser scanning confocal microscope (FluoView-1000, Olympus) coupled to an inverted microscope IX-81(Olympus). Images were obtained with an Olympus UPLSAPO 60X W NA: 1.2 and zoom x2 (800 × 800 pixels, 0.13 μm/pixel). Samples were excited with 488/500–530 nm excitation/emission filters for EdU staining, 543/555–625 nm for bacteria producing DsRed and 633/LP650 nm for Topro-3 nuclear staining. Multiple fluorescence signals were acquired sequentially to avoid cross-talk between colour channels. For 3D acquisition, optical sectioning of the specimen (Z series) was driven by a Z-axis stepping motor and maximum intensity projection was further generated.

### Extraction and purification of RNA

1 × 10^6^ cells of *A. castellanii* were infected with *L. pneumophila* Paris at a MOI of 20 for 2 h in 6-well plates. After the infection (0 h), cells were harvested for RNA extraction at different time points 2 h, 16 h and 24 h. To increase the efficiency of the cell lysis, RNA extractions were preceded by a mechanical cell lysis using a FastPrep^®^-24 Instrument (MP) for 30 seconds at a speed of 5.0 (twice) with FastPrep Tubes containing glass breads 150–212 μm (Sigma). RNA extraction was performed using the High Pure RNA Isolation Kit (Roche).

An additional digestion of residual DNA was performed with Turbo DNA-free^™^ kit (Ambion^®^) according to manufacturer’s instructions.

### Reverse transcription (RT) and quantitative PCR (qPCR)

Reverse transcription of RNA was performed using GoScript^™^ Reverse Transcription System (Promega) following manufacturer’s recommendations. Products of reverse transcription (RT) were purified with NucleoSpin Gel and PCR Clean-up kit (Macherey-Nagel). All cDNA were normalized (10 μg/mL) before proceeding to qRT-PCR reaction. All qPCR reactions were performed using the LightCycler^®^ FastStart DNA Master^Plus^ SYBR Green I kit (Roche) on the LightCycler 1.5 instrument (Roche). Each tube contained 9 μL of reaction mix containing 0.5 μL of each primers CDC2b (XM_004353658.1) (5′ATGCAAGCCAAACCCAGTC 3′ and reverse: 5′ GAATCGCTGGTTCTCGGTATC 3′) or 18S primers^51^ at 10 μM, 2 μL of Master Mix 5X, 6 μL of water and 1 μL of cDNA or 1 μL of water for negative control. The qPCR program was: 10 min at 95 °C and 45 cycles of the amplification step (10 sec at 95 °C, 10 sec at 61 °C and 10 sec at 72 °C for extension time in a single acquisition mode), the melting curves step for 1 min at 65 °C for annealing and the cooling 30 seconds at 40 °C. The relative level of genes expression was calculated using the 2^−∆∆Ct^ method^52^. Expression level were normalized to the 18S mRNA.

### Phylogenetic analysis

To construct the phylogenetic tree, proteins were aligned using MUSCLE of the software MEGA6 with a Maximum likelihood and a bootstrap replications of 1000.

### *A. castellanii* CDC2b cloning

The *CDC2b* (XM_004353658.1) cDNA from *A. castellanii* was amplified by PCR from total cDNA with flanking BamHI and XbaI restriction sites using primers ACA-cdc2b-5′ (TTTTTTTGGATCCTACACA**ATG**CAAGCCAAACCCAGTCC) and ACA-cdc2b-3′-STOP (TTTTTTTTCTAGATTAGGGGTACTTGGTCTTGTCC) and inserted between BamHI and XbaI sites of the *S. cerevisiae* expression plasmid pYES2-CT (Invitrogen), yielding pWA-cdc2b-Ac. Plasmid DNA was prepared with a NucleoSpin Plasmid kit (Macherey-Nagel).

Yeast transformation of Y838 was done by the Li Acetate method as described by Gietz and Woods^53^. This strain is temperature sensitive for growth (ts^−^) due to a deficient *CDC28* gene product. Transformants were selected at the permissive temperature (28 °C) on YNB medium supplemented with adequate amino acids and bases. For complementation assays of the *cdc28-4*ts^−^ phenotype, transformants were streaked on YPGal medium and incubated at the indicated temperature (30 °C, 34 °C or 37 °C) for 4 days.

### Statistical analyses

All experiments were performed three times. Results were analyzed by Two-way RM ANOVA with the Bonferroni post-test (GraphPad Prism5) or using a one-tailed Mann-Whitney test considering a statistical significance at p ≤ 0.05. All data are average of three independents experiments and error bars represent the standard error of the mean (±SEM).

## Additional Information

**How to cite this article**: Mengue, L. *et al*. Legionella *pneumophila* prevents proliferation of its natural host *Acanthamoeba castellanii. Sci. Rep.* **6**, 36448; doi: 10.1038/srep36448 (2016).

**Publisher’s note**: Springer Nature remains neutral with regard to jurisdictional claims in published maps and institutional affiliations.

## Supplementary Material

Supplementary Video 1

Supplementary Video 2

Supplementary Video Legends

Supplementary Data 1

## Figures and Tables

**Table 1:** Putative *A. castellanii* CDK resulting from the blast of all human CDKs^18^.

Accession number	Name
XP_004353710.1	Cell division control protein 2b, putative (CDC2b)
XP_004336625.1	Cdk10/11, putative
XP_004367653.1	Protein kinase domain containing protein
XP_004335644.1	Cyclindependent kinase
XP_004342572.1	Protein kinase domain containing protein
