# Supplementary material for: Legionella pneumophila prevents proliferation of its natural host Acanthamoeba castellanii
Source: Sci Rep. 2016 Nov 2;6:36448. doi: 10.1038/srep36448 (PMC5091012; doi:10.1038/srep36448)
Supplement: Supplementary Video Legends [file srep36448-s3.pdf]

Legionella pneumophila prevents proliferation of its natural host *Acanthamoeba castellanii*

Luce Mengue, Matthieu Régnacq, Willy Aucher, Emilie Portier, Yann Héchard, Ascel Samba-Louaka

Legend for supplementary Information

Video 1: *A. castellanii* motility and cell division.

A. castellanii 30010 were visualized by a time lapse microscopy experiment. The movie record ran around 17 h. Cells seemed highly motile and some cytokinesis figures were observed.

Video 2: *L. pneumophila* prevents cell division of *A. castellanii.*

A. castellanii ATCC 30010 were infected with *L. pneumophila* Paris expressing GFP (G_EP10) at a MOI of 20. No cell division was observed with GFP positive cells. The movie record started 16 h after the end of the infection and ran for around 17 h.
