# Supplementary material for: Legionella pneumophila prevents proliferation of its natural host Acanthamoeba castellanii
Source: Sci Rep. 2016 Nov 2;6:36448. doi: 10.1038/srep36448 (PMC5091012; doi:10.1038/srep36448)
Supplement: Supplementary Data 1 [file srep36448-s4.pdf]

Legionella pneumophila prevents proliferation of its natural host *Acanthamoeba castellanii*

Luce Mengue, Matthieu Régnacq, Willy Aucher, Emilie Portier, Yann Héchard, Ascel Samba-Louaka

Supplementary Data 1

Homo sapiens Cyclin-Dependent Kinase (CDK) protein sequences

>CDK1

MEDYTKIEKIGEGTYGVVYKGRHKTTGQVVAMKKIRLESEEEGVPSTAIREISLLKEL
RHPNIVSLQDVLMQDSRLYLIFEFLSMDLKKYLDSIPPGQYMDSSLVKSYLYQILQGI
VFCHSRRVLHRDLKPQNLLIDDKGTIKLADFGLARAFGIPIRVYTHEVVTLWYRSPEV
LLGSARYSTPVDIWSIGTIFAELATKKPLFHGDSEIDQLFRIFRALGTPNNEVWPEVESL
QDYKNTFPKWKPGSLASHVKNLDENGLDLLSKMLIYDPAKRISGKMALNHPYFNDL
DNQIKKM

>CDK2

MENFQKVEKIGEGTYGVVYKARNKLTGEVVALKKIRLDTETEGVPSTAIREISLLKEL
NHPNIVKLLDVIHTENKLYLVF

EFLHQDLKKFMDASALTGIPLPLIKSYLFQLLQGLAFCHSHRVLHRDLKPQNLLINTE
GAIKLADFGLARAFGVPVRTYTHEVVTLWYRAPEILLGCKYYSTAVDIWSLGCIFAE
MVTRRALFPGDSEIDQLFRIFRTLGTPDEVVWPGVTSMPDYKPSFPKWARQDFSKVV
PPLDEDGRSLLSQMLHYDPNKRISAKAALAHPFFQDVTKPVPHLRL

>CDK3

MDMFQKVEKIGEGTYGVVYKAKNRETGQLVALKKIRLDLEMEGVPSTAIREISLLKE

LKHPNIVRLLDVVHNERKLYLVFEFLSQDLKKYMDSTPGSELPLHLIKSYLFQLLQGV

SFCHSHRVIHRDLKPQNLLINELGAIKLADFGLARAFGVPLRTYTHEVVTLWYRAPEI

LLGSKFYTTAVDIWSIGCIFAEMVTRKALFPGDSEIDQLFRIFRMLGTPSEDTWPGVTQ

LPDYKGSFPKWTRKGLEEIVPNLEPEGRDLLMQLLQYDPSQRITAKTALAHPYFSSPE

PSPAARQYVLQRFRH

>CDK4

MATSRYEPVAEIGVGAYGTVYKARDPHSGHFVALKSVRVPNGGGGGGGLPISTVRE

VALLRRLEAFEHPNVVRLMDVCATSRTDREIKVTLVFEHVDQDLRTYLDKAPPPGLP

AETIKDLMRQFLRGLDFLHANCIVHRDLKPENILVTSGGTVKLADFGLARIYSYQMA

LTPVVVTLWYRAPEVLLQSTYATPVDMWSVGCIFAEMFRRKPLFCGNSEADQLGKIF

DLIGLPPEDDWPRDVSLPRGAFPPRGPRPVQSVVPEMEESGAQLLLEMLTFNPHKRIS

AFRALQHSYLHKDEGNPE

> CDK5

MQKYEKLEKIGEGTYGTVFKAKNRETHEIVALKRVRLDDDDEGVPSSALREICLLKE

LKHKNIVRLHDVLHSDKKLTLVFEFCDQDLKKYFDSCNGDLDPEIVKSFLFQLLKGL

GFCHSRNVLHRDLKPQNLLINRNGELKLADFGLARAFGIPVRCYSAEVVTLWYRPPD

VLFGAKLYSTSIDMWSAGCIFAELANAGRPLFPGNDVDDQLKRIFRLLGTPTEEQWPS

MTKLPDYKPYPMYPATTSLVNVVPKLNATGRDLLQNLLKCNPVQRISAEEALQHPYF

SDFCPP

>CDK6

MEKDGLCRADQQYECVAEIGEGAYGKVFKARDLKNGGRFVALKRVRVQTGEEGMP

LSTIREVAVLRHLETFEHPNVVRLFDVCTVSRTDRETKLTLVFEHVDQDLTTYLDKVP

EPGVPTETIKDMMFQLLRGLDFLHSHRVVHRDLKPQNILVTSSGQIKLADFGLARIYS

FQMALTSVVVTLWYRAPEVLLQSSYATPVDLWSVGCIFAEMFRRKPLFRGSSDVDQL

GKILDVIGLPGEEDWPRDVALPRQAFHSKSAQPIEKFVTDIDELGKDLLLKCLTFNPA

KRISAYSALSHPYFQDLERCKENLDSHLPPSQNTSELNTA

>CDK7

MALDVKSRAKRYEKLDFLGEGQFATVYKARDKNTNQIVAIKKIKLGHRSEAKDGIN

RTALREIKLLQELSHPNIIGLLDAFGHKSNISLVFDFMETDLEVIIKDNSLVLTPSHIKA

YMLMTLQGLEYLHQHWILHRDLKPNNLLLDENGVLKLADFGLAKSFGSPNRAYTHQ

VVTRWYRAPELLFGARMYGVGVDMWAVGCILAELLLRVPFLPGDSDLDQLTRIFET

LGTPTEEQWPDMCSLPDYVTFKSFPGIPLHHIFSAAGDDLLDLIQGLFLFNPCARITAT

QALKMKYFSNRPGPTPGCQLPRPNCPVETLKEQSNPALAIKRKRTEALEQGGLPKKLI

F

>CDK9

MAKQYDSVECPFCDEVSKYEKLAKIGQGTFGEVFKARHRKTGQKVALKKVLMENE

KEGFPITALREIKILQLLKHENVVNLIEICRTKASPYNRCKGSIYLVFDFCEHDLAGLLS

NVLVKFTLSEIKRVMQMLLNGLYYIHRNKILHRDMKAANVLITRDGVLKLADFGLA

RAFSLAKNSQPNRYTNRVVTLWYRPPELLLGERDYGPPIDLWGAGCIMAEMWTRSPI

MQGNTEQHQLALISQLCGSITPEVWPNVDNYELYEKLELVKGQKRKVKDRLKAYVR

DPYALDLIDKLLVLDPAQRIDSDDALNHDFFWSDPMPSDLKGMLSTHLTSMFEYLAP
PRRKGSQITQQSTNQSRNPATTNQTEFERVF

>CDK10

MAEPDLECEQIRLKCIRKEGFFTVPPEHRLGRCRSVKEFEKLNRIGEGTYGIVYRARDT
QTDEIVALKKVRMDKEKDGIPISSLREITLLLRLRHPNIVELKEVVVGNHLESIFLVMG
YCEQDLASLLENMPTPFSEAQVKCIVLQVLRGLQYLHRNFII

HRDLKVSNLLMTDKGCVKTADFGLARAYGVPVKPMTPKVVTLWYRAPELLLGTTT
QTTSIDMWAVGCILAELLAHRPLLPGTSEIHQIDLIVQLLGTPSENIWPGFSKLPLVGQ
YSLRKQPYNNLKHKFPWLSEAGLRLLHFLFMYDPKKRATAGDCLESSYFKEKPLPCE
PELMPTFPHHRNKRAAPATSEGQSKRCKP

>CDK11a

MGDEKDSWKVKTLDEILQEKKRRKEQEEKAEIKRLKNSDDRDSKRDSLEEGELRDH
CMEITIRNSPYRREDSMEDRGEEDDSLAIKPPQQMSRKEKVHHRKDEKRKEKCRHHS
HSAEGGKHARVKEREHERRKRHREEQDKARREWERQKRREMAREHSRRERDRLEQ
LERKRERERKMREQQKEQREQKERERRAEERRKEREARREVSAHHRTMREDYSDKV
KASHWSRSPPRPPRERFELGDGRKPVKEEKMEERDLLSDLQDISDSERKTSSAESSSA
ESGSGSEEEEEEEEEEEEEGSTSEESEEEEEEEEEEEEETGSNSEEASEQSAEEVSEEEM
SEDEERENENHLLVVPESRFDRDSGESEEAEEEVGEGTPQSSALTEGDYVPDSPALLPI
ELKQELPKYLPALQGCRSVEEFQCLNRIEEGTYGVVYRAKDKKTDEIVALKRLKMEK
EKEGFPITSLREINTILKAQHPNIVTVREIVVGSNMDKIYIVMNYVEHDLKSLMETMK
QPFLPGEVKTLMIQLLRGVKHLHDNWILHRDLKTSNLLLSHAGILKVGDFGLAREYG
SPLKAYTPVVVTQWYRAPELLLGAKEYSTAVDMWSVGCIFGELLTQKPLFPGNSEID
QINKVFKELGTPSEKIWPGYSELPVVKKMTFSEHPYNNLRKRFGALLSDQGFDLMNK

FLTYFPGRRISAEDGLKHEYFRETPLPIDPSMFPTWPAKSEQQRVKRGTSPRPPEGGLG
YSQLGDDDLKETGFHLTTTNQGASAAGPGFSLKF

>CDK11b

MGDEKDSWKVKTLDEILQEKKRRKEQEEKAEIKRLKNSDDRDSKRDSLEEGELRDH
RMEITIRNSPYRREDSMEDRGEEDDSLAIKPPQQMSRKEKVHHRKDEKRKEKRRHRS
HSAEGGKHARVKEKEREHERRKRHREEQDKARREWERQKRREMAREHSRRERDRL
EQLERKRERERKMREQQKEQREQKERERRAEERRKEREARREVSAHHRTMREDYSD
KVKASHWSRSPPRPPRERFELGDGRKPVKEEKMEERDLLSDLQDISDSERKTSSAESS
SAESGSGSEEEEEEEEEEEEEEGSTSEESEEEEEEEEEEEEEETGSNSEEASEQSAEEVSEEE
MSEDEERENENHLLVVPESRFDRDSGESEEAEEEVGEGTPQSSALTEGDYVPDSPALS
PIELKQELPKYLPALQGCRSVEEFQCLNRIEEGTYGVVYRAKDKKTDEIVALKRLKM
EKEKEGFPITSLREINTILKAQHPNIVTVREIVVGSNMDKIYIVMNYVEHDLKSLMETM
KQPFLPGEVKTLMIQLLRGVKHLHDNWILHRDLKTSNLLLSHAGILKVGDFGLAREY
GSPLKAYTPVVVTLWYRAPELLLGAKEYSTAVDMWSVGCIFGELLTQKPLFPGKSEI
DQINKVFKDLGTPSEKIWPGYSELPAVKKMTFSEHPYNNLRKRFGALLSDQGFDLMN
KFLTYFPGRRISAEDGLKHEYFRETPLPIDPSMFPTWPAKSEQQRVKRGTSPRPPEGGL
GYSQLGDDDLKETGFHLTTTNQGASAAGPGFSLKF

>CDK12

MPNSERHGGKKDGSGGASGTLQPSSGGGSSNSRERHRLVSKHKRHKSKHSKDMGLV
TPEAASLGTVIKPLVEYDDISSDSDTFSDDMAFKLDRRENDERRGSDRSDRLHKHRH
HQHRRSRDLLKAKQTEKEKSQEVSSKSGSMKDRISGSSKRSNEETDDYGKAQVAKSS
SKESRSSKLHKEKTRKERELKSGHKDRSKSHRKRETPKSYKTVDSPKRRSRSPHRKW
SDSSKQDDSPSGASYGQDYDLSPSRSHTSSNYDSYKKSPGSTSRRQSVSPPYKEPSAY
QSSTRSPSPYSRRQRSVSPYSRRRSSSYERSGSYSGRSPSPYGRRRSSSPFLSKRSLSRSP

LPSRKSMKSRSRSPAYSRHSSSHSKKKRSSSRSRHSSISPVRLPLNSSLGAELSRKKKER
AAAAAAAKMDGKESKGSPVFLPRKENSSVEAKDSGLESKKLPRSVKLEKSAPDTELV
NVTHLNTEVKNSSDTGKVKLDENSEKHLVKDLKAQGTRDSKPIALKEEIVTPKETETS
EKETPPPLPTIASPPPPLPTTTPPPQTPPLPPLPPIPALPQQPPLPPSQPAFSQVPASSTSTL
PPSTHSKTSAVSSQANSQPPVQVSVKTQVSVTAAIPHLKTSTLPPLPLPPLLPGDDDMD
SPKETLPSKPVKKEKEQRTRHLLTDLPLPPELPGGDLSPPDSPEPKAITPPQQPYKKRP
KICCPRYGERRQTESDWGKRCVDKFDIIGIIGEGTYGQVYKAKDKDTGELVALKKVR
LDNEKEGFPITAIREIKILRQLIHRSVVNMKEIVTDKQDALDFKKDKGAFYLVFEYMD
HDLMGLLESGLVHFSEDHIKSFMKQLMEGLEYCHKKNFLHRDIKCSNILLNNSGQIKL
ADFGLARLYNSEESRPYTNKVITLWYRPPELLLGEERYTPAIDVWSCGCILGELFTKK
PIFQANLELAQLELISRLCGSPCPAVWPDVIKLPYFNTMKPKKQYRRRLREEFSFIPSA
ALDLLDHMLTLDPSKRCTAEQTLQSDFLKDVELSKMAPPDLPHWQDCHELWSKKRR
RQRQSGVVVEEPPPSKTSRKETTSGTSTEPVKNSSPAPPQPAPGKVESGAGDAIGLADI
TQQLNQSELAVLLNLLQSQTDLSIPQMAQLLNIHSNPEMQQQLEALNQSISALTEATS
QQQDSETMAPEESLKEAPSAPVILPSAEQTTLEASSTPADMQNILAVLLSQLMKTQEP
AGSLEENNSDKNSGPQGPRRTPTMPQEEAAACPPHILPPEKRPPEPPGPPPPPPPPLVE
GDLSSAPQELNPAVTAALLQLLSQPEAEPPGHLPHEHQALRPMEYSTRPRPNRTYGNT
DGPETGFSAIDTDERNSGPALTESLVQTLVKNRTFSGSLSHLGESSSYQGTGSVQFPGD
QDLRFARVPLALHPVVGQPFLKAEGSSNSVVHAETKLQNYGELGPGTTGASSSGAGL
HWGGPTQSSAYGKLYRGPTRVPPRGGRGRGVPY

>CDK13

MPSSSDTALGGGGGLSWAEKKLEERRKRRRFLSPQQPPLLLPLLQPQLLQPPPPPPPLL
FLAAPGTAAAAAAAAAASSSCFSPGPPLEVKRLARGKRRAGGRQKRRRGPRAGQEA
EKRRVFSLPQPQQDGGGGASSGGGVTPLVEYEDVSSQSEQGLLLGGASAATAATAA
GGTGGSGGSPASSSGTQRRGEGSERRPRRDRRSSSGRSKERHREHRRRDGQRGGSEA

SKSRSRHSHSGEERAEVAKSGSSSSSGGRRKSASATSSSSSSRKDRDSKAHRSRTKSSK

EPPSAYKEPPKAYREDKTEPKAYRRRRSLSPLGGRDDSPVSHRASQSLRSRKSPSPAG

GGSSPYSRRLPRSPSPYSRRRSPSYSRHSSYERGGDVSPSPYSSSSWRRSRSPYSPVLRR

SGKSRSRSPYSSRHSRSRSRHRLSRSRSRHSSISPSTLTLKSSLAAELNKNKKARAAEA

ARAAEAAKAAEATKAAEAAAKAAKASNTSTPTKGNTETSASASQTNHVKDVKKIKI

EHAPSPSSGGTLKNDKAKTKPPLQVTKVENNLIVDKATKKAVIVGKESKSAATKEES

VSLKEKTKPLTPSIGAKEKEQHVALVTSTLPPLPLPPMLPEDKEADSLRGNISVKAVK

KEVEKKLRCLLADLPLPPELPGGDDLSKSPEEKKTATQLHSKRRPKICGPRYGETKEK

DIDWGKRCVDKFDIIGIIGEGTYGQVYKARDKDTGEMVALKKVRLDNEKEGFPITAIR

EIKILRQLTHQSIINMKEIVTDKEDALDFKKDKGAFYLVFEYMDHDLMGLLESGLVHF

NENHIKSFMRQLMEGLDYCHKKNFLHRDIKCSNILLNNRGQIKLADFGLARLYSSEES

RPYTNKVITLWYRPPELLLGEERYTPAIDVWSCGCILGELFTKKPIFQANQELAQLELI

SRICGSPCPAVWPDVIKLPYFNTMKPKKQYRRKLREEFVFIPAAALDLFDYMLALDPS

KRCTAEQALQCEFLRDVEPSKMPPPDLPLWQDCHELWSKKRRRQKQMGMTDDVSTI

KAPRKDLSLGLDDSRTNTPQGVLPSSQLKSQGSSNVAPVKTGPGQHLNHSELAILLNL

LQSKTSVNMADFVQVLNIKVNSETQQQLNKINLPAGILATGEKQTDPSTPQQESSKPL

GGIQPSSQTIQPKVETDAAQAAVQSAFAVLLTQLIKAQQSKQKDVLLEERENGSGHE

ASLQLRPPPEPSTPVSGQDDLIQHQDMRILELTPEPDRPRILPPDQRPPEPPEPPPVTEED

LDYRTENQHVPTTSSSLTDPHAGVKAALLQLLAQHQPQDDPKREGGIDYQAGDTYV

STSDYKDNFGSSSFSSAPYVSNDGLGSSSAPPLERRSFIGNSDIQSLDNYSTASSHSGGP

PQPSAFSESFPSSVAGYGDIYLNAGPMLFSGDKDHRFEYSHGPIAVLANSSDPSTGPES

THPLPAKMHNYNYGGNLQENPSGPSLMHGQTWTSPAQGPGYSQGYRGHISTSTGRG

RGRGLPY

>CDK14

MHGYFGCNAAAEPGYSAFVGTPQICVTKMSTRNCQGMDSVIKPLDTIPEDKKVRVQ

RTQSTFDPFEKPANQVKRVHSENNACINFKTSSTGKESPKVRRHSSPSSPTSPKFGKAD

SYEKLEKLGEGSYATVYKGKSKVNGKLVALKVIRLQEEEGTPFTAIREASLLKGLKH

ANIVLLHDIIHTKETLTLVFEYVHTDLCQYMDKHPGGLHPDNVKLFLFQLLRGLSYIH

QRYILHRDLKPQNLLISDTGELKLADFGLARAKSVPSHTYSNEVVTLWYRPPDVLLGS

TEYSTCLDMWGVGCIFVEMIQGVAAFPGMKDIQDQLERIFLVLGTPNEDTWPGVHSL

PHFKPERFTLYSSKNLRQAWNKLSYVNHAEDLASKLLQCSPKNRLSAQAALSHEYFS

DLPPRLWELTDMSSIFTVPNVRLQPEAGESMRAFGKNNSYGKSLSNSKH

>CDK15

MTSFHPRGLQAARAQKFKSKRPRSNSDCFQEEDLRQGFQWRKSLPFGAASSYLNLEK

LGEGSYATVYKGISRINGQLVALKVISMNAEEGVPFTAIREASLLKGLKHANIVLLHDI

IHTKETLTFVFEYMHTDLAQYMSQHPGGLHPHNVRLFMFQLLRGLAYIHHQHVLHR

DLKPQNLLISHLGELKLADFGLARAKSIPSQTYSSEVVTLWYRPPDALLGATEYSSEL

DIWGAGCIFIEMFQGQPLFPGVSNILEQLEKIWEVLGVPTEDTWPGVSKLPNYNPEWF

PLPTPRSLHVVWNRLGRVPEAEDLASQMLKGFPRDRVSAQEALVHDYFSALPSQLYQ

LPDEESLFTVSGVRLKPEMCDLLASYQKGHHPAQFSKCW

>CDK16

MDRMKKIKRQLSMTLRGGRGIDKTNGAPEQIGLDESGGGGGSDPGEAPTRAAPGELR

SARGPLSSAPEIVHEDLKMGSDGESDQASATSSDEVQSPVRVRMRNHPPRKISTEDIN

KRLSLPADIRLPEGYLEKLTLNSPIFDKPLSRRLRRVSLSEIGFGKLETYIKLDKLGEGT

YATVYKGKSKLTDNLVALKEIRLEHEEGAPCTAIREVSLLKDLKHANIVTLHDIIHTE

KSLTLVFEYLDKDLKQYLDDCGNIINMHNVKLFLFQLLRGLAYCHRQKVLHRDLKP
QNLLINERGELKLADFGLARAKSIPTKTYSNEVVTLWYRPPDILLGSTDYSTQIDMWG
VGCIFYEMATGRPLFPGSTVEEQLHFIFRILGTPTEETWPGILSNEEFKTYNYPKYRAE
ALLSHAPRLDSDGADLLTKLLQFEGRNRISAEDAMKHPFFLSLGERIHKLPDTTSIFAL
KEIQLQKEASLRSSSMPDSGRPAFRVVDTEF

>CDK17

MKKFKRRLSLTLRGSQTIDESLSELAEQMTIEENSSKDNEPIVKNGRPPTSHSMHSFLH
QYTGSFKKPPLRRPHSVIGGSLGSFMAMPRNGSRLDIVHENLKMGSDGESDQASGTS
SDEVQSPTGVCLRNRIHRRISMEDLNKRLSLPADIRIPDGYLEKLQINSPPFDQPMSRRS
RRASLSEIGFGKMETYIKLEKLGEGTYATVYKGRSKLTENLVALKEIRLEHEEGAPCT
AIREVSLLKDLKHANIVTLHDIVHTDKSLTLVFEYLDKDLKQYMDDCGNIMSMHNV
KLFLYQILRGLAYCHRRKVLHRDLKPQNLLINEKGELKLADFGLARAKSVPTKTYSN
EVVTLWYRPPDVLLGSSEYSTQIDMWGVGCIFFEMASGRPLFPGSTVEDELHLIFRLL
GTPSQETWPGISSNEEFKNYNFPKYKPQPLINHAPRLDSEGIELITKFLQYESKKRVSA
EEAMKHVYFRSLGPRIHALPESVSIFSLKEIQLQKDPGFRNSSYPETGHGKNRRQSMLF

>CDK18

MIMNKMKNFKRRFSLSVPRTETIEESLAEFTEQFNQLHNRRNENLQLGPLGRDPPQEC
STFSPTDSGEEPGQLSPGVQFQRRQNQRRFSMEVRASGALPRQVAGCTHKGVHRRAA
ALQPDFDVSKRLSLPMDIRLPQEFLQKLQMESPDLPKPLSRMSRRASLSDIGFGKLET
YVKLDKLGEGTYATVFKGRSKLTENLVALKEIRLEHEEGAPCTAIREVSLLKNLKHA
NIVTLHDLIHTDRSLTLVFEYLDSDLKQYLDHCGNLMSMHNVKIFMFQLLRGLAYCH
HRKILHRDLKPQNLLINERGELKLADFGLARAKSVPTKTYSNEVVTLWYRPPDVLLG
STEYSTPIDMWGVGCIHYEMATGRPLFPGSTVKEELHLIFRLLGTPTEETWPGVTAFS

EFRTYSFPCYLPQPLINHAPRLDTDGIHLLSSLLLYESKSRMSAEAALSHSYFRSLGER

VHQLEDTASIFSLKEIQLQKDPGYRGLAFQQPGRGKNRRQSIF

>CDK19

MDYDFKAKLAAERERVEDLFEYEGCKVGRGTYGHVYKARRKDGKDEKEYALKQIE

GTGISMSACREIALLRELKHPNVIALQKVFLSHSDRKVWLLFDYAEHDLWHIIKFHRA

SKANKKPMQLPRSMVKSLLYQILDGIHYLHANWVLHRDLKPANILVMGEGPERGRV

KIADMGFARLFNSPLKPLADLDPVVVTFWYRAPELLLGARHYTKAIDIWAIGCIFAEL

LTSEPIFHCRQEDIKTSNPFHHDQLDRIFSVMGFPADKDWEDIRKMPEYPTLQKDFRR

TTYANSSLIKYMEKHKVKPDSKVFLLLQKLLTMDPTKRITSEQALQDPYFQEDPLPTL

DVFAGCQIPYPKREFLNEDDPEEKGDKNQQQQNQHQQPTAPPQQAAAPPQAPPPQ

QNSTQTNGTAGGAGAGVGGTGAGLQHSQDSSLNQVPPNKKPRLGPSGANSGGPVMP

SDYQHSSSRLNYQSSVQGSSQSQSTLGYSSSSQQSSQYHPSHQAHRY

>CDK20

MDQYCILGRIGEGAHGIVFKAKHVETGEIVALKKVALRRLEDGFPNQALREIKALQE

MEDNQYVVQLKAVFPHGGGFVLAFEFMLSDLAEVVRHAQRPLAQAQVKSYLQMLL

KGVAFCHANNIVHRDLKPANLLISASGQLKIADFGLARVFSPDGSRLYTHQVATRWY

RAPELLYGARQYDQGVDLWSVGCIMGELLNGSPLFPGKNDIEQLCYVLRILGTPNPQ

VWPELTELPDYNKISFKEQVPMPLEEVLPDVSPQALDLLGQFLLYPPHQRIAASKALL

HQYFFTAPLPAHPSELPIPQRLGGPAPKAHPGPPHIHDFHVDRPLEESLLNPELIRPFILE

G

Homo sapiens outgroup protein sequences used to built the phylogenetic tree

>CDKL1

MMEKYEKIGKIGEGSYGVVFKCRNRDTGQIVAIKKFLESEDDPVIKKIALREIRMLKQ
LKHPNLVNLLEVFRRKRRLHLVFEYCDHTVLHELDRYQRGVPEHLVKSITWQTLQA
VNFCHKHNCIHRDVKPENILITKHSVIKLCDFGFARLLTGPSDYYTDYVATRWYRSPE
LLVGDTQYGPPVDVWAIGCVFAELLSGVPLWPGKSDVDQLYLIRKTLGDLIPRHQQV
FSTNQYFSGVKIPDPEDMEPLELKFPNISYPALGLLKGCLHMDPTQRLTCEQLLHHPY
FENIREIEDLAKEHNKPTRKTLRKSRKHHCFTETSKLQYLPQLTGSSILPALDNKKYYC
DTKKLNYRFPNI

>GSK3alpha

MSGGGPSGGGPGGSGRARTSSFAEPGGGGGGGGGGPGGSASGPGGTGGGKASVGA
MGGGVGASSSGGGPGGSGGGGSGGPGAGTSFPPPGVKLGRDSGKVTTVVATLGQGP
ERSQEVAYTDIKVIGNGSFGVVYQARLAETRELVAIKKVLQDKRFKNRELQIMRKLD
HCNIVRLRYFFYSSGEKKDELYLNLVLEYVPETVYRVARHFTKAKLTIPILYVKVYM
YQLFRSLAYIHSQGVCHRDIKPQNLLVDPDTAVLKLCDFGSAKQLVRGEPNVSYICSR
YYRAPELIFGATDYTSSIDVWSAGCVLAELLLGQPIFPGDSGVDQLVEIIKVLGTPTRE
QIREMNPNYTEFKFPQIKAHPWTKVFKSRTPPEAIALCSSLLEYTPSSRLSPLEACAHSF
FDELRCLGTQLPNNRPLPPLFNFSAGELSIQPSLNAILIPPHLRSPAGTTTLTPSSQALTE
TPTSSDWQSTDATPTLTNSS

>MAK

MNRYTTMRQLGDGTYGSVLMGKSNESGELVAIKRMKRKFYSWDECMNLREVKSLK

KLNHANVIKLKEVIRENDHLYFIFEYMKENLYQLMKDRNKLFPESVIRNIMYQILQGL

AFIHKHGFFHRDMKPENLLCMGPELVKIADFGLARELRSQPPYTDYVSTRWYRAPEV

LLRSSVYSSPIDVWAVGSIMAELYMLRPLFPGTSEVDEIFKICQVLGTPKKSDWPEGY

QLASSMNFRFPQCVPINLKTLIPNASNEAIQLMTEMLNWDPKKRPTASQALKHPYFQ

VGQVLGPSSNHLESKQSLNKQLQPLESKPSLVEVEPKPLPDIIDQVVGQPQPKTSQQPL

QPIQPPQNLSVQQPPKQQSQEKPPQTLFPSIVKNMPTKPNGTLSHKSGRRRWGQTIFKS

GDSWEELEDYDFGASHSKKPSMGVFKEKRKKDSPFRLPEPVPSGSNHSTGENKSLPA

VTSLKSDSELSTAPTSKQYYLKQSRYLPGVNPKKVSLIASGKEINPHTWSNQLFPKSL

GPVGAELAFKRSNAGNLGSYATYNQSGYIPSFLKKEVQSAGQRIHLAPLNATASEYT

WNTKTGRGQFSGRTYNPTAKNLNIVNRAQPIPSVHGRTDWVAKYGGHR

Putative *A. castellanii* CDK resulting from the blast of all human CDKs

>XP_004353710.1 Cdc2b

MQAKPSPLQRYDIKEKLGEGTYGEVYKAIDTENQRFIALKKMRLLEAEDEGVPATAL

REVSLLKELSNCANIVKLLDVIHCNSTLYLVFEFLDQDLKTYVESTGAGALPTKLVKS

YLYQILKGIAYCHSHRILHRDLKLANLLIDRKGVLKLADFGLARAFGVPIRTYTHEVV

TLWYRAPEILLGQARYSTPVDMWSVGCIFAELVTKRPLFPGDCEIDELFRIFRTLGTPN

EEVWPGVTTLPDYKSTFGQWKPQSLASVVPGLDPLGLDLLSKMLRYAPQERISAKEA

LKHPYFDDLDKTKYP

>XP_004336625.1 cdk10/11

MEDQQQPSRPRGRGPIVGLSPPSGSTKRRAHDAEDEHTPPAVGTKRARSSSGGKEREE

GGATPTAGNGLPASPAPRAPSPSSSSSSSSSVPPKGASPGQRAASPQGTPLRAAAAGLP

VLVAAMRSCRSVDHYERLNRIQEGAYGVVHRAKDKETGEVVALKKFKIKGDESFPV

TALRELAVLMEMDHPNVVRAKEIVIGKDPNSFYLVMEFLEHDLKDLMTAMRDPFLQ

SEIKCLLQQLLEAIAYIHDNWYLHRDLKTSNLLLSSKGILKVADFGLARHYGDPLRPY

TQPVVTLWYLAKRPIDPYRAPELLLGAKEYSWEIDMWAVGCIFAEMLCKEPLMKAQ

TELQMIDQIFKMLGTPNDDVWPGFSELPFVKKMKFKTYPSAIRQRMFSATTDAGFDL

LMSFLAYDPKKRITAREALSHRYFTEPPLPREPGMIQTFPSLHEGTRPRPNSPGVEGGL

FGAHLLSDSTDAHAALGIDPYGHQPTGFKLRF

>XP_004367653.1 protein kinase domain containing protein

MKRSREEMPTTLHRRTQSHGSILPTNASSSSSSARASTFLPPAAPSLGSSSSSSSSVARP

PSHIQITRPPQPRRETSSRHTPTPTSGGRLAANPSTPAAAKSSSSPFGGFSASRAIDESLL

MGTCRRIQQYETLEQVGEGTYGVVYRARDMRSNRIVALKKVRMEKEKDGIPITCLRE

VKILKEVDHPNIVRLLGVTVGRQLDAMYLAFEYVEHDLAGLIDNMKKPFTEAEVKC
LMMQLLRAVHYLHRNYYIHRDLKLSNLLFSNKGELKLCDFGLARIFGEPNKAMTPK
VDMWAVGCIFGELLMNTPVMPGKTEIEQLQLMCRLLGTPNDKIWPGYYELPNAKIIA
GLPQQNYHMISNKFKFLSPAGQDLIKKFLTYDPNKRITAYQALQHPYFTESPMPKSPD
MMPTFPTKLNTGYRRARRSDEVQEDRRMDEERRHQNDRFGEVFGDHDYAGGAPLK
KRRF

>XP_004335644.1 Cyclindependent kinase

MTIFKKPAVGLRQRLTFDDFHIIEQVGEGTYGRVFKARNKHTNKLTALKVVFPTEDD
EGLPFTAVREIKYLQMLHDNPNVIKLEGTFFTKDGELVLAFEYMENDLSGLLSLKNL
QFTPAQTKCLFKQVLEGLHQCHSAGIMHRDIKAANLLLNNGQLKLADFGLASNYAR
RRTFSTNVVTLWYRAPELLLGVNTYGPKVDIWSAGCLFIELLTRQSPFPGREEKHQLE
LIVRTCGTPDERNWPGVTKLEGYKLLQGLMGHKNRLSEVFGKFDPRALDLLSKMLA
LNPAQRPTASEALDHDYFWADPLPCKATELPHYPAMHEYEAKKTRQNERQPKRQKV
SNYAPNPYPSHHHQGYPSGAPAPQSSRGDYAPPPHYPHPSYPSKPGQTLLWVLTGDP
RLTERIPKPTGTPPLRAMAFSRRLPSLFPPPTRCGRPATQALVDSQAGMRLRGQWVRG
HLSITVAPLLHRRPCSETANTPPRPATPAQLPPAGRSAEYSRVPREHRPPTSAAMAPSS
LGGGGHHLGGPRHDMPPMASYNRPPAGAHRPHHHLHHHQQLHHPITDGHASAAAY
SGVHRSHARATLSSVERMEVGVDTMGKRKREAAVPL

>XP_004342572.1 protein kinase domain containing protein

MIFKKPAVHVQRLSFDDFYIIEQVGEGTYGRVFKARNVHTNKLTALKVVFPTEDDEG
LPFTAVREIKYLQMLSDNPNVIKLEGTFFTRDGELVLAFEYMENDLSGLLSLKNLQFS
PAQTKCLFKQVLEGLHQCHRAGIMHRDIKAANLLLNNGELKMADFGLASNYLRRRT
FSTNVVTLWYRAPELLLGVNAYGPKVDIWSAGCLFIELLTRQSPFPGREEKHQLELIV
RTCGTPDERNWPGVTKLEGYKQLQGLMGHKNRLREVFGKFDPRALDLLSRMLSLNP

ADRPTASEALDHDYFWTDPVPCKATDLPHYPAMHEYEAKKTRQSERQPKRQRVTGY

APNVPVAPLRSQPYPPHNAGYPPHVPLPVPQQPARGDYPPPLPSPHYAHLPYPSSKLG

QYGQPPARQNLPTGPQGWGPAAHGTHPPAAGNGLLPTPHVPLTVPGHPPRPTGYAG

ASSYPGIHPPSAMPHYDAARSSGSRHRQPSSGSLVASARHPLVSSGLPPHRIGGAQPQS

SSSSSSLPPSTGLRGGYHHAGPREMNRPPTSSARVEGGPRHDMPPHLASGYNRPPAGA

HRPVHHHAALQHHNHLGHPIIADGHHAPTAHNSVHKSHARATLSSVERMGVGVDTT

GKRKREATPLS
